# Tight junction protein ZO-1 in Kawasaki disease

**DOI:** 10.1186/s12887-021-02622-2

**Published:** 2021-03-31

**Authors:** Wan-Tz Lai, Hung-Chang Lee, Ying-Hsien Huang, Mao-Hung Lo, Ho-Chang Kuo

**Affiliations:** 1 Department of Pediatric Gastroenterology, Kaohsiung Chang Gung Memorial Hospital, Kaohsiung, Taiwan; 2 Department of Pediatrics, Chiayi Chang Gung Memorial Hospital, Chiayi, Taiwan; 3 Department of Pediatric Gastroenterology, Hepatology, and Nutrition, MacKay Children’s Hospital, Taipei, Taiwan; 4 Department of Pediatrics, Kaohsiung Chang Gung Memorial Hospital and Chang Gung University College of Medicine, Kaohsiung, Taiwan; 5 Kawasaki Disease Center, Kaohsiung Chang Gung Memorial Hospital, #123 Da-Pei Road, Niaosong District, Kaohsiung, 83301 Taiwan

**Keywords:** Kawasaki disease, Tight junction protein, Coronary artery lesions

## Abstract

**Background:**

Kawasaki disease (KD) is a form of systemic febrile vasculitis that is complicated with coronary artery lesions (CAL). The tight junctions that maintain the intestinal barrier also play a role in systemic inflammatory diseases. Serum zonula occludens-1 (ZO-1) expression was found to be significantly lower in asthmatic patients, and another study reported that elevated systemic ZO-1 was positively correlated with inflammation in cirrhotic patients. A murine model of KD vasculitis demonstrated that vasculitis depended on intestinal barrier dysfunction, which is maintained by tight junctions. In this study, we aimed to investigate the role of the tight junction zonula occludens-1 (ZO-1) in the treatment response of intravenous immunoglobulin (IVIG) and the occurrence of CAL formation in KD patients.

**Methods:**

We enrolled 40 KD patients, 12 healthy controls, and 12 febrile controls in this study. The serum levels of tight junction ZO-1 were determined by enzyme-linked immunosorbent assay.

**Results:**

The serum ZO-1 level was higher in the fever control group but did not reach a statistical significance. KD patients who received a second dose of IVIG treatment due to initial IVIG unresponsiveness had a higher serum levels of tight junction ZO-1, but without statistical significance (2.15 ± 0.18 vs. 2.69 ± 0.31 ng/mL, *p* = 0.058). KD patients who developed a CAL demonstrated a significant lower serum tight junction ZO-1 levels than KD without CAL formation (1.89 ± 0.16 vs. 2.39 ± 0.15 ng/mL, *p* = 0.027). After multiple logistic regression analysis, ZO-1 levels [(95% confidence interval (CI): 0.058 ~ 0.941, odds ratio (OR) = 0.235, *p* = 0.041)] showed as the risk factor for CAL formation.

**Conclusion:**

Serum levels of tight junction ZO-1 levels were lower in KD patients than fever controls and associated with CAL formation.

## Background

Kawasaki disease (KD) is an acute febrile coronary vasculitis disease that primarily occurs in children under the age of 5 years old. First described by Dr. Tomisaku Kawasaki in 1967, the first report of Kawasaki disease in English was published in 1974 [1]. The clinical presentation of complete Kawasaki disease patients includes prolonged fever (more than 5 days) and at least four of the following five major symptoms: cervical lymphadenopathy, bilateral non-purulent conjunctivitis, indurative angioedema of the hands and feet, diffuse mucosal inflammation, and polymorphous skin rashes [2]. Cardiovascular involvement such as fistula formation [3], coronary artery lesions (CAL), coronary artery dilatation and coronary artery aneurysm (CAA), are major complications of KD [4, 5].

KD also affects the mucosal intestinal immune responses, and KD patients have an increased number of activated T cells and macrophages in the small intestine [6]. A recent multicenter study of more than 300 patients revealed that abdominal and gastrointestinal symptoms at KD onset can complicate KD diagnosis, cause therapeutic delay, and increase the risk for IVIG resistance and coronary aneurysms [7, 8]. Furthermore, Rivas et al. demonstrated that a murine model of KD vasculitis depended on intestinal barrier dysfunction, which led to secretory IgA leakage and IgA-C3 immune complex deposition in cardiovascular lesions [8]. Zonula occludens (ZO)-1 is a multi-domain polypeptide required for the assembly of tight junctions [9] and thus essential for the intestinal barrier.

Recent studies showed that ZO-1 was also associated with inflammation. ZO-1 expression levels were significantly lower in asthmatic patients and may have played a role in inflammation [10]. Another study reported that elevated systemic ZO-1 was positively correlated with inflammation in cirrhotic patients [11]. Kawasaki disease is also a systemic febrile inflammatory disease. However, no studies have investigated the role of the tight junction ZO-1 protein in the development of KD vasculitis. The purpose of this study was to observe the serum ZO-1 levels in KD patients and evaluate the possible association between ZO-1 expression and the disease outcome of KD.

## Methods

### Patients

According to the American Heart Association [12], the diagnosis of Kawasaki disease can be established using the clinical criteria of fever for ≥5 days (body temperature ≥ 38 °C) and the presence of ≥4 of the 5 following principal clinical features: cervical lymphadenopathy, bilateral non-purulent conjunctivitis, indurative angioedema of the hands and feet, diffuse mucosal inflammation, and polymorphous skin rashes [2]. In this case control study, we enrolled 40 patients who fulfilled the complete criteria of KD (blood samples were collected 24 h before IVIG treatment), 12 healthy controls, and 12 febrile controls. KD patients who were treated pursuant to the American Heart Association’s recommendations [5] of one dose of intravenous immunoglobulin (dosage: 2 g/kg) in a 12-h period. The Institutional Review Board of Chang Gung Memorial Hospital approved the study with registry number 102-5947C. We obtained written informed consent from the parents or guardians of all patients. Patients whose symptoms did not meet the diagnostic criteria of Kawasaki disease were excluded. Coronary artery lesion (CAL) was defined as a coronary artery internal diameter greater than 3 mm (or 4 mm if the patient was more than 5 years old) or of a segment greater than 1.5 times the inner diameter of an adjacent segment exanimated in echocardiography [13, 14]. The definition of patient responsiveness to IVIG treatment was patients’ body temperature within a normal range for 48 h, without recurrence of fever for more than 7 days after completing IVIG treatment, as well as the clinical improvement of inflammatory signs [15, 16]. Febrile control patients were admitted because of upper and/or lower respiratory tract infections, such as acute pharyngitis/tonsillitis, croup, and acute bronchiolitis/bronchitis. Blood samples from the febrile controls were used for comparison. The blood samples were immediately placed in tubes containing heparin, and the remaining serum aliquots were stored at − 80 °C until assay.

### Measurement of ZO-1 by enzyme-linked immunoassay (ELISA)

We followed the manufacturer’s instructions to measure ZO-1 (Aviva Systems Biology, OKDD00562) using enzyme-linked immunoassays (ELISA).

### Statistical analysis

The data are presented as mean ± standard deviation. We adopted student’s t-test (for comparison of 2 variances) or one-way analysis of variance (ANOVA, for comparison of KD, fever control and health control) to analyze the quantitative data. Multivariate analysis with logistic regression were used to assess the parameters of CAL risk in KD. Statistical significance was considered as two-sided *p*-values less than 0.05. We used SPSS version 13.0 for Windows XP (SPSS, Inc., Chicago, USA) to perform all the statistical tests.

## Results

We enrolled 12 non-fever healthy control (HC) subjects, 12 fever control (FC) patients (patients with fever but without a history of KD or diagnosed as having KD), and 40 KD patients in this study. No significant difference was observed in age or sex between the non-fever healthy control, fever control, and KD groups. All the KD patients met the AHA 2004 diagnostic criteria (complete KD) [5].

**Fig. 1:**
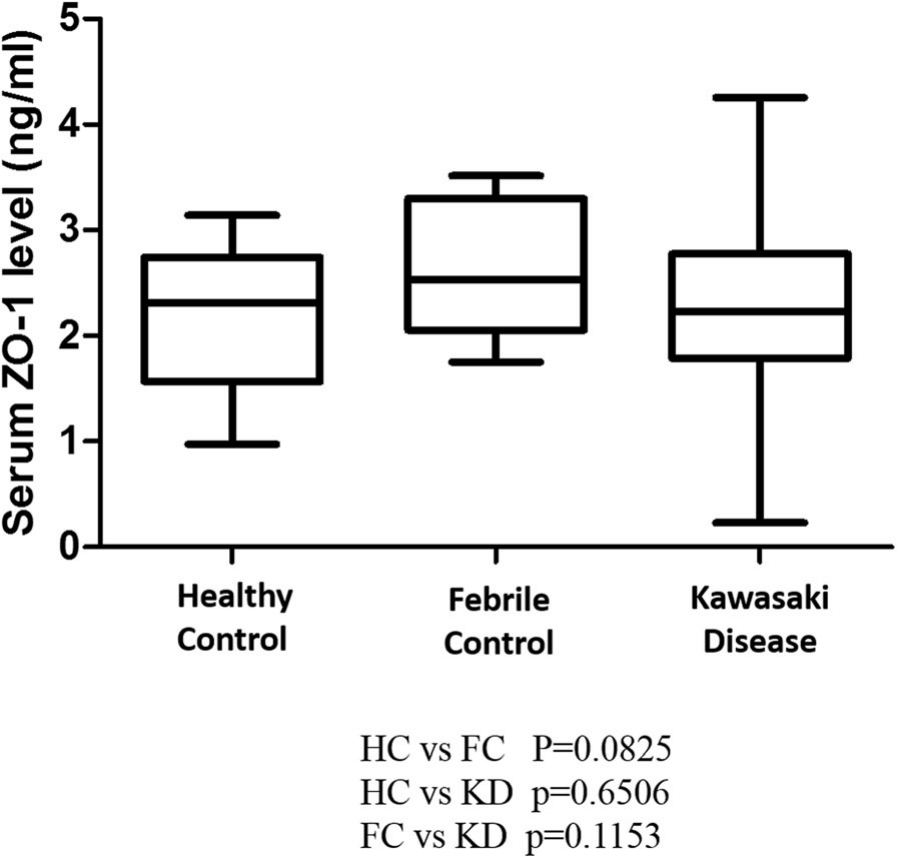
ZO-1 protein expression is determined by ELISA. We enrolled 40 patients with KD, 12 healthy controls, and 12 febrile controls in this study

The demographic data of HC, FC and KD were showed in Table 1. (age, sex, fever duration, CAL and IVIG resistance) There were no significant difference in regarding age and sex in HC, FC and KD. (*p* > 0.05) KD patients had higher fever during than FC group (*p* = 0.03). There were 8 cases with IVIG resistance and 21 cases with CAL formation in KD in this case control study. The average serum tight junction ZO-1 level of each group was 2.15 ± 0.19 ng/mL in the HC group, 2.63 ± 0.18 ng/mL in the FC group, and 2.26 ± 0.12 ng/mL in the KD group. The fever control group had a higher serum ZO-1 level, but did not reach statistical significance (HC vs. FC, *P* = 0.08; HC vs. KD, *p* = 0.65; FC vs. KD, *p* = 0.11) (Fig. 1).

**Table 1:** Baseline characteristics of patients with KD and controls

Characteristic	Healthy controls (*n* = 12)	Febrile controls (*n* = 12)	KD (*n* = 40)	*p* value
Male sex	4/12 (33.3%)	5/12 (41.7%)	20/40 (50%)	0.573
Age (year, range)	2.04 ± 0.12	2.87 ± 0.41	1.87 ± 0.25	0.108
	(1.4–2.7)	(1.0–4.6)	(0.3–8.5)	
Fever days		5.75 ± 0.90	8.48 ± 0.61	0.269
WBC (1000/uL)	8.4 ± 0.8	8.0 ± 0.7	13.2 ± 1.0	0.001*
RBC (million/Ul)	4.7 ± 0.1	4.4 ± 0.1	4.4 ± 0.1	0.182
Hemoglobin (g/dL)	12.5 ± 0.3	11.7 ± 0.3	11.3 ± 0.1	0.001*
CRP (mg/L)		61.0 ± 15.7	73.9 ± 12.7	0.074
CAL formation			21/40 (52.5%)	
IVIG resistance			8/40 (20%)	

*CAL* coronary artery lesion, *IVIG* intravenous immunoglobulin, *KD* Kawasaki disease, *CRP* C-Reactive protein, *WBC* white blood cell, *RBC* red blood cell

Data expressed as mean ± SD

* indicate p value < 0.05

**Fig. 2:**
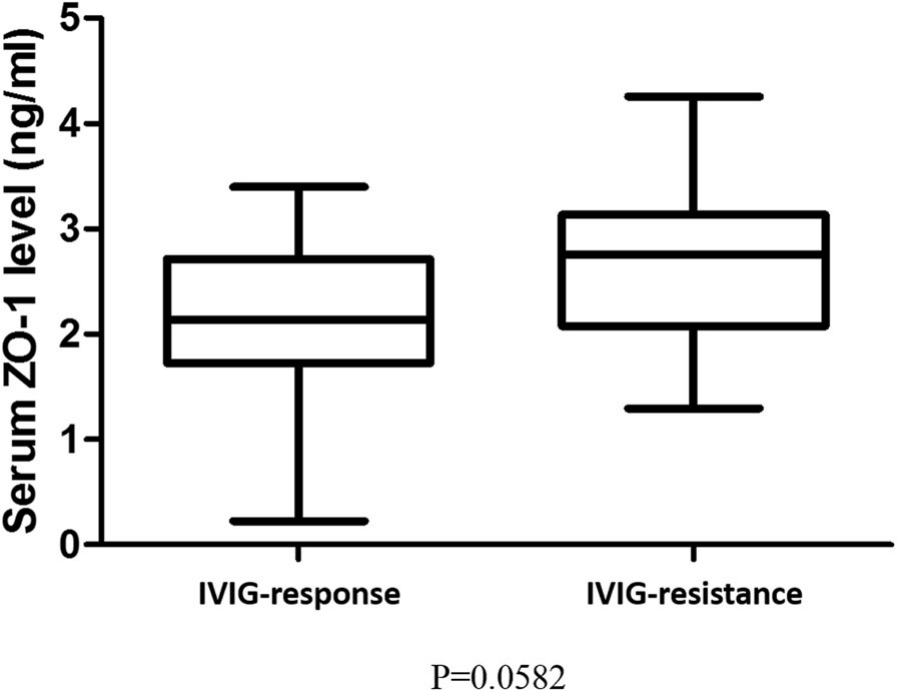
There is no significant difference in ZO-1 protein between intravenous immunoglobulin responsiveness or resistance in Kawasaki disease patients

**Fig. 3:**
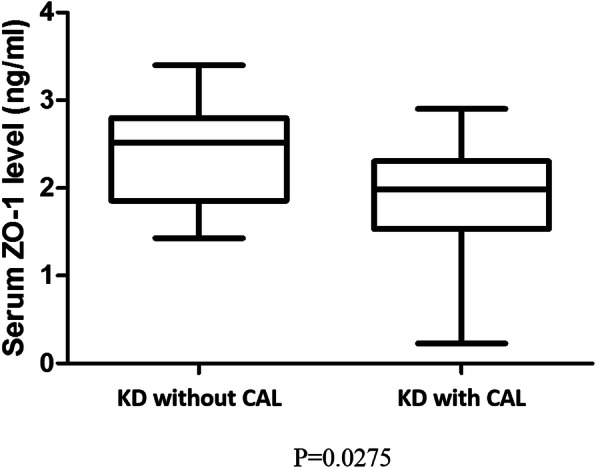
The ZO-1 protein is higher in Kawasaki disease patients with coronary arterial lesion. *p < 0.05. Data are presented as mean ± standard deviation

We classified Kawasaki disease patients into two groups: IVIG responsive (*N* = 32) and IVIG resistant (*N* = 8). As shown in Fig. 2, the IVIG-resistant group had a higher serum ZO-1 level, but did not reach statistical significance (2.15 ± 0.12 vs. 2.69 ± 0.31 ng/mL, *p* = 0.058). In the KD group, which consisted of a total of 40 patients, after removed 8 cases with IVIG resistance, there were 16/32 (50%) patients had CAL for further analysis. The average Z-score of CAL patients was 3.04 ± 0.92. The finding indicates that the CAL group had a significant lower serum levels of tight junction ZO-1 level than without CAL group (1.89 ± 0.16 vs. 2.40 ± 0.15 ng/mL, *p* = 0.027) (Fig. 3). After multiple logistic regression analysis, ZO-1 levels [(95% confidence interval (CI): 0.058 ~ 0.941, odds ratio (OR) = 0.235, *p* = 0.041)] showed as the risk factor for CAL formation, but not found in regarding fever duration (95% CI: 0.909 ~ 1.291, OR = 1.08, *p* = 0.37), IVIG resistance (95% CI: 0.340 ~ 8.175, OR = 1.67, *p* = 0.53), and age (95% CI: 0.74 ~ 1.67, OR = 1.11, *p* = 0.61).

## Discussion

Dr. Tomisaku Kawasaki of Japan first prescribed a case series of Kawasaki disease patients in 1967 [1]. The first case of KD in Taiwan was described in 1976 [17]. The prevalence of KD is highest in Japan [18] and lowest among children of European descent [19]. Although the incidence of Kawasaki disease has been steadily increasing worldwide [20], a definitive disease pathogenesis remains uncertain. A practical biomarker may help physicians to stratify therapy for KD according to the likelihood of developing CAL. Some studies have reported that the tight junction ZO-1 protein was related to some inflammatory diseases and was correlated with the inflammatory marker. Ram et al. reported that hepatocellular carcinoma patients have significantly increased serum ZO-1 levels, which has a positive correlation with inflammatory markers [21]. To the best of our knowledge, our study is the first to survey the correlation of tight junction ZO-1 protein expression in KD patients.

In one KD mice model, TNF-α levels rapidly increased during Lactobacillus-cell wall extract (LCWE)-induced coronary arteritis, as well as were essential to inducing coronary artery inflammation and aneurysm formation [22]. Rochfort reported TNF-αdose-dependent reduced ZO-1 expression in brain endothelium [23]. In our study, lower serum ZO-1 levels were noted in the CAL group among KD patients. Therefore, we may interpret that the serum ZO-1 level has a negative correlation with the development of coronary arterial lesions in humans.

In one mice KD study [24], Lactobacillus-cell wall extract (LCWE)-injected mice developed KD vasculitis and exhibited a significant increase in intestinal leakage. The intestinal barrier dysfunction was associated with decreased Zo-1 expression [25]. Kawasaki disease also affects mucosal intestinal immune responses, and KD patients demonstrated an increased amount of activated macrophages and T cells in the small intestine [6, 8]. New evidence has suggested that strong development of immune responses may lead to endothelial dysfunction, which is also essential for CAL development in Kawasaki disease patients [16, 26, 27].

About 20% of KD patients presented with various gastrointestinal manifestations, such as abdominal distension or pain, vomiting, diarrhea, jaundice, paralytic ileus, hepatomegaly, gallbladder hydrops, and related echographic findings [5, 28, 29]. A recent multicenter study that enrolled more than 300 patients revealed that Kawasaki disease patients with gastrointestinal presentations at the onset of the disease had a complicated KD diagnosis, therapeutic delay, or risk for IVIG-unresponsiveness and coronary aneurysms [7]. Our results showed no significant difference between groups of IVIG responsiveness and resistance in KD patients; however, a similar difference in a slightly larger sample size would have likely resulted in a statistical significance, and further studies are needed. Future research may survey the association between serum ZO-1 levels and IVIG responsiveness in additional large-size studies.

To the best of our understanding, this study was the first to investigate the association between Kawasaki disease and tight junction Zo-1 level. However, the limitation of this study is that the direct correlation between Zo-1 concentration in serum and coronary or intestinal tissue has not been studied in KD patients. This required larger scale of study or further investigation.

## Conclusions

In conclusion, tight junction ZO-1 levels decrease in KD patients with coronary artery lesions. Tight junction ZO-1 may participate in the intestinal barrier dysfunction of KD and may be a potential serum marker for predicting CAL in KD patients. The pathophysiological basis of these findings in KD warrants further investigation.

## Data Availability

The datasets used and/or analyzed during the current study are not publicly available for ethical and privacy reasons, but are available from the corresponding author on reasonable request.

## Abbreviations

KD: Kawasaki disease
CAL: Coronary artery lesion
CAA: Coronary artery aneurysm
ZO: Zonula occludens
IVIG: Intravenous immunoglobulin
TJs: Tight junctions
HC: Healthy control
LCWE: Lactobacillus-cell wall extract
